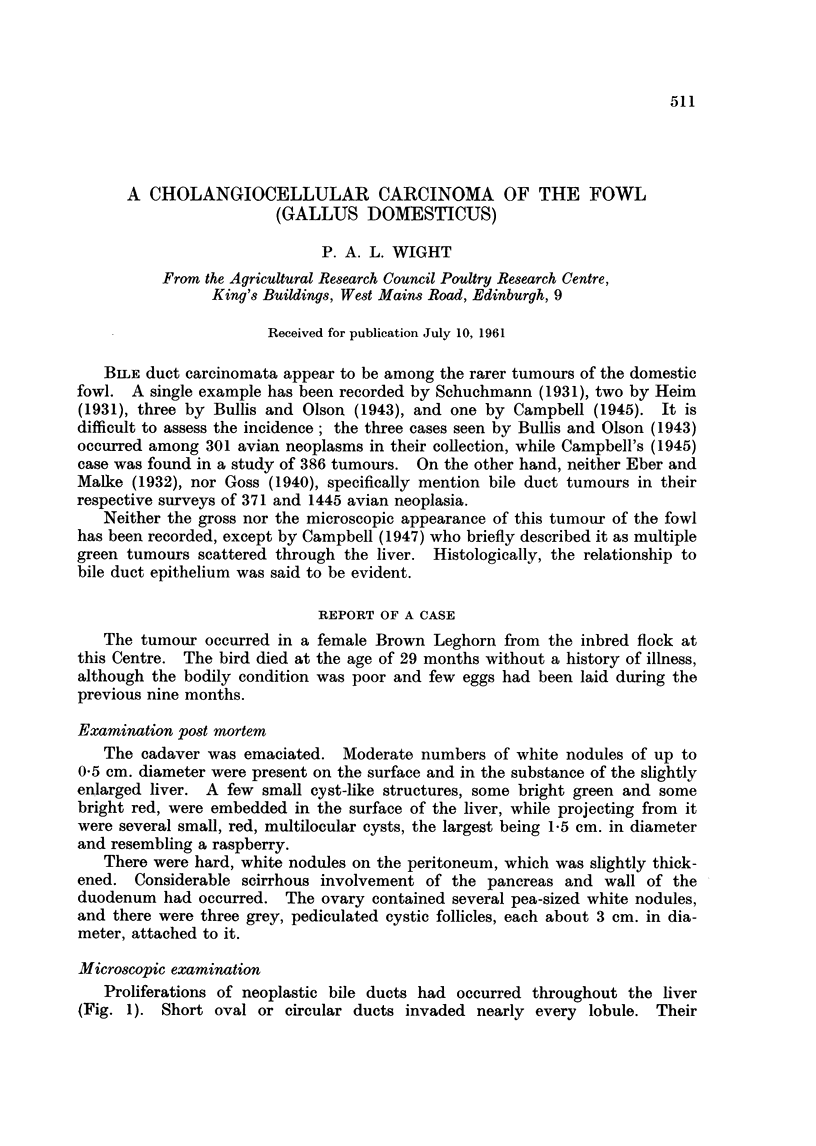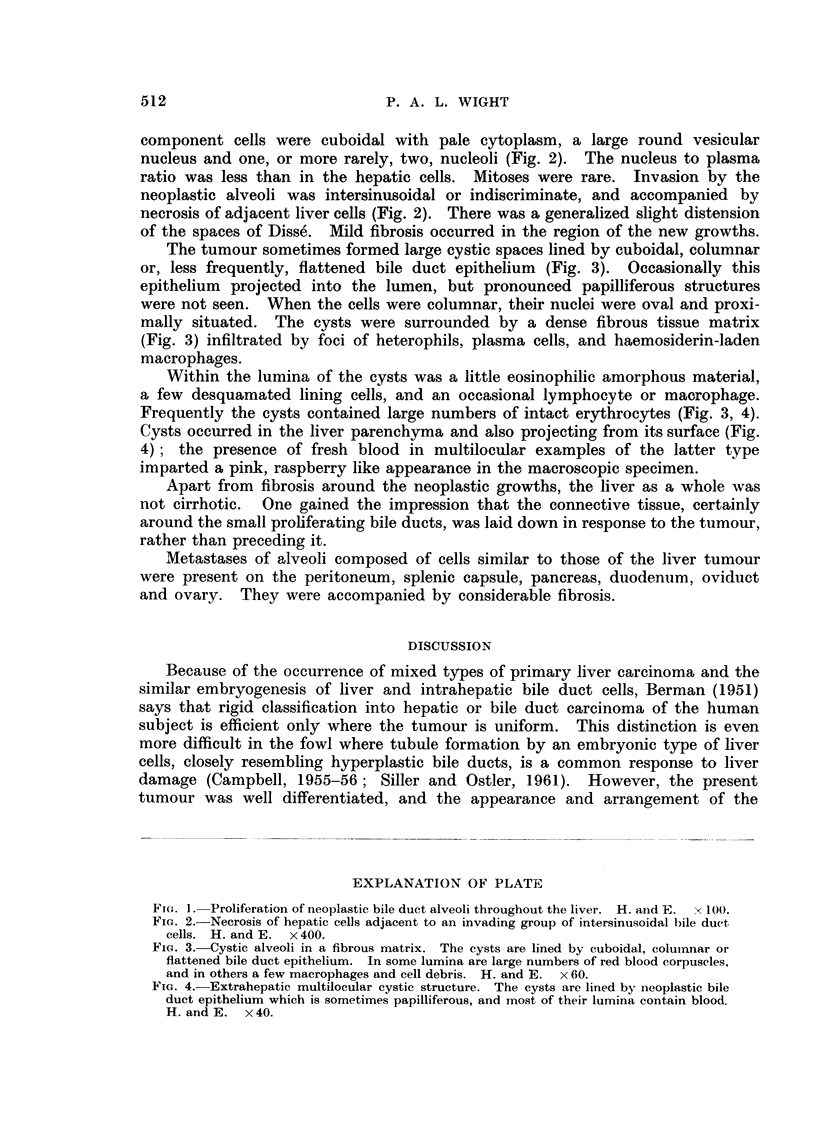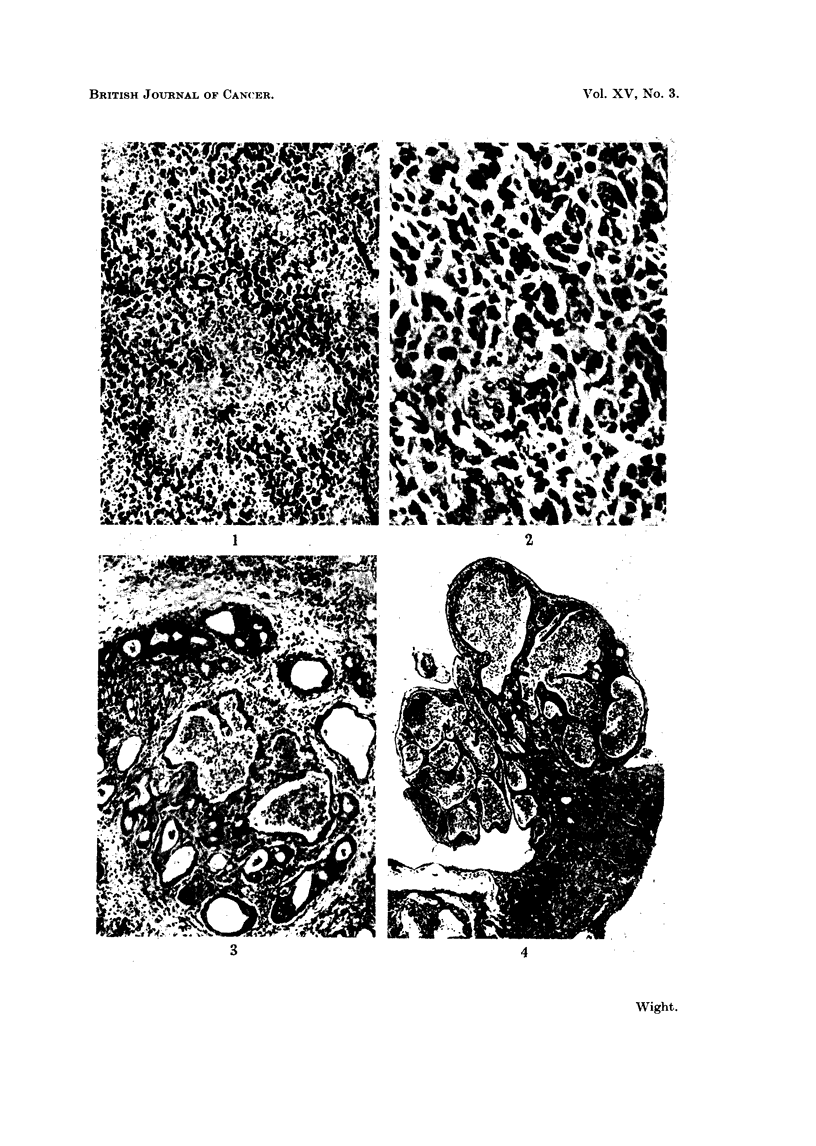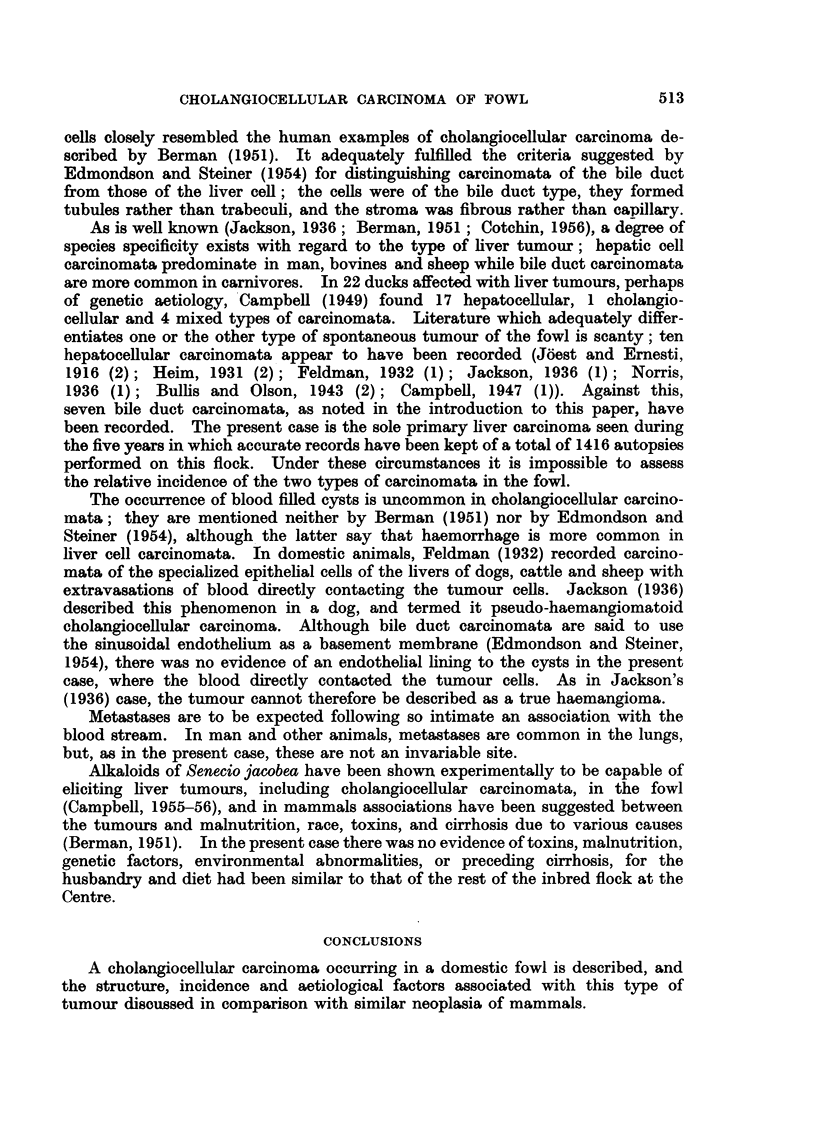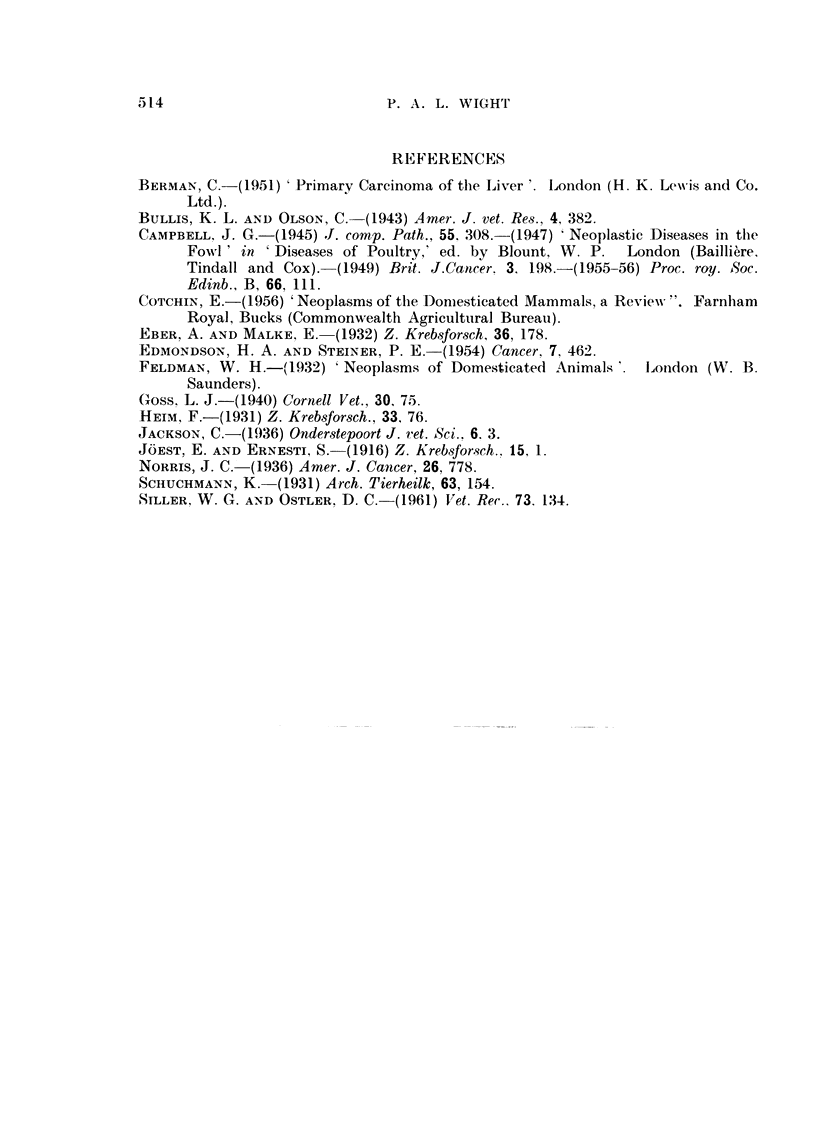# A Cholangiocellular Carcinoma of the Fowl (Gallus Domesticus)

**DOI:** 10.1038/bjc.1961.61

**Published:** 1961-09

**Authors:** P. A. L. Wight

## Abstract

**Images:**


### A CHOLANGIOCELLULAR CARCINOMA OF THE FOWL (GALLUS DOMESTICUS)

P. A. L. WIGHT

*From the Agricultural Research Council Poultry Research Centre, King's Buildings, West Mains Road, Edinburgh, 9*

Received for publication July 10, 1961

BILE duct carcinomata appear to be among the rarer tumours of the domestic fowl. A single example has been recorded by Schuchmann (1931), two by Heim (1931), three by Bullis and Olson (1943), and one by Campbell (1945). It is difficult to assess the incidence ; the three cases seen by Bullis and Olson (1943) occurred among 301 avian neoplasms in their collection, while Campbell's (1945) case was found in a study of 386 tumours. On the other hand, neither Eber and Malke (1932), nor Goss (1940), specifically mention bile duct tumours in their respective surveys of 371 and 1445 avian neoplasia.

Neither the gross nor the microscopic appearance of this tumour of the fowl has been recorded, except by Campbell (1947) who briefly described it as multiple green tumours scattered through the liver. Histologically, the relationship to bile duct epithelium was said to be evident.

### REPORT OF A CASE

The tumour occurred in a female Brown Leghorn from the inbred flock at this Centre. The bird died at the age of 29 months without a history of illness, although the bodily condition was poor and few eggs had been laid during the previous nine months.

### *Examination post mortem*

The cadaver was emaciated. Moderate numbers of white nodules of up to 0·5 cm. diameter were present on the surface and in the substance of the slightly enlarged liver. A few small cyst-like structures, some bright green and some bright red, were embedded in the surface of the liver, while projecting from it were several small, red, multilocular cysts, the largest being 1·5 cm. in diameter and resembling a raspberry.

There were hard, white nodules on the peritoneum, which was slightly thickened. Considerable scirrhous involvement of the pancreas and wall of the duodenum had occurred. The ovary contained several pea-sized white nodules, and there were three grey, pediculated cystic follicles, each about 3 cm. in diameter, attached to it.

### *Microscopic examination*

Proliferations of neoplastic bile ducts had occurred throughout the liver (Fig. 1). Short oval or circular ducts invaded nearly every lobule. Their component cells were cuboidal with pale cytoplasm, a large round vesicular nucleus and one, or more rarely, two, nucleoli (Fig. 2). The nucleus to plasma ratio was less than in the hepatic cells. Mitoses were rare. Invasion by the neoplastic alveoli was intersinusoidal or indiscriminate, and accompanied by necrosis of adjacent liver cells (Fig. 2). There was a generalized slight distension of the spaces of Dissé. Mild fibrosis occurred in the region of the new growths.

The tumour sometimes formed large cystic spaces lined by cuboidal, columnar or, less frequently, flattened bile duct epithelium (Fig. 3). Occasionally this epithelium projected into the lumen, but pronounced papilliferous structures were not seen. When the cells were columnar, their nuclei were oval and proximally situated. The cysts were surrounded by a dense fibrous tissue matrix (Fig. 3) infiltrated by foci of heterophils, plasma cells, and haemosiderin-laden macrophages.

Within the lumina of the cysts was a little eosinophilic amorphous material, a few desquamated lining cells, and an occasional lymphocyte or macrophage. Frequently the cysts contained large numbers of intact erythrocytes (Fig. 3, 4). Cysts occurred in the liver parenchyma and also projecting from its surface (Fig. 4) ; the presence of fresh blood in multilocular examples of the latter type imparted a pink, raspberry like appearance in the macroscopic specimen.

Apart from fibrosis around the neoplastic growths, the liver as a whole was not cirrhotic. One gained the impression that the connective tissue, certainly around the small proliferating bile ducts, was laid down in response to the tumour, rather than preceding it.

Metastases of alveoli composed of cells similar to those of the liver tumour were present on the peritoneum, splenic capsule, pancreas, duodenum, oviduct and ovary. They were accompanied by considerable fibrosis.

### DISCUSSION

Because of the occurrence of mixed types of primary liver carcinoma and the similar embryogenesis of liver and intrahepatic bile duct cells, Berman (1951) says that rigid classification into hepatic or bile duct carcinoma of the human subject is efficient only where the tumour is uniform. This distinction is even more difficult in the fowl where tubule formation by an embryonic type of liver cells, closely resembling hyperplastic bile ducts, is a common response to liver damage (Campbell, 1955–56 ; Siller and Ostler, 1961). However, the present tumour was well differentiated, and the appearance and arrangement of the

---

### EXPLANATION OF PLATE

Fig. 1.—Proliferation of neoplastic bile duct alveoli throughout the liver. H. and E. ×100.

Fig. 2.—Necrosis of hepatic cells adjacent to an invading group of intersinusoidal bile duct cells. H. and E. ×400.

Fig. 3.—Cystic alveoli in a fibrous matrix. The cysts are lined by cuboidal, columnar or flattened bile duct epithelium. In some lumina are large numbers of red blood corpuscles, and in others a few macrophages and cell debris. H. and E. ×60.

Fig. 4.—Extrahepatic multilocular cystic structure. The cysts are lined by neoplastic bile duct epithelium which is sometimes papilliferous, and most of their lumina contain blood. H. and E. ×40.

Wight.

cells closely resembled the human examples of cholangiocellular carcinoma described by Berman (1951). It adequately fulfilled the criteria suggested by Edmondson and Steiner (1954) for distinguishing carcinomata of the bile duct from those of the liver cell; the cells were of the bile duct type, they formed tubules rather than trabeculi, and the stroma was fibrous rather than capillary.

As is well known (Jackson, 1936; Berman, 1951; Cotchin, 1956), a degree of species specificity exists with regard to the type of liver tumour; hepatic cell carcinomata predominate in man, bovines and sheep while bile duct carcinomata are more common in carnivores. In 22 ducks affected with liver tumours, perhaps of genetic aetiology, Campbell (1949) found 17 hepatocellular, 1 cholangiocellular and 4 mixed types of carcinomata. Literature which adequately differentiates one or the other type of spontaneous tumour of the fowl is scanty; ten hepatocellular carcinomata appear to have been recorded (Jöest and Ernesti, 1916 (2); Heim, 1931 (2); Feldman, 1932 (1); Jackson, 1936 (1); Norris, 1936 (1); Bullis and Olson, 1943 (2); Campbell, 1947 (1)). Against this, seven bile duct carcinomata, as noted in the introduction to this paper, have been recorded. The present case is the sole primary liver carcinoma seen during the five years in which accurate records have been kept of a total of 1416 autopsies performed on this flock. Under these circumstances it is impossible to assess the relative incidence of the two types of carcinomata in the fowl.

The occurrence of blood filled cysts is uncommon in cholangiocellular carcinomata; they are mentioned neither by Berman (1951) nor by Edmondson and Steiner (1954), although the latter say that haemorrhage is more common in liver cell carcinomata. In domestic animals, Feldman (1932) recorded carcinomata of the specialized epithelial cells of the livers of dogs, cattle and sheep with extravasations of blood directly contacting the tumour cells. Jackson (1936) described this phenomenon in a dog, and termed it pseudo-haemangiomatoid cholangiocellular carcinoma. Although bile duct carcinomata are said to use the sinusoidal endothelium as a basement membrane (Edmondson and Steiner, 1954), there was no evidence of an endothelial lining to the cysts in the present case, where the blood directly contacted the tumour cells. As in Jackson's (1936) case, the tumour cannot therefore be described as a true haemangioma.

Metastases are to be expected following so intimate an association with the blood stream. In man and other animals, metastases are common in the lungs, but, as in the present case, these are not an invariable site.

Alkaloids of *Senecio jacobea* have been shown experimentally to be capable of eliciting liver tumours, including cholangiocellular carcinomata, in the fowl (Campbell, 1955–56), and in mammals associations have been suggested between the tumours and malnutrition, race, toxins, and cirrhosis due to various causes (Berman, 1951). In the present case there was no evidence of toxins, malnutrition, genetic factors, environmental abnormalities, or preceding cirrhosis, for the husbandry and diet had been similar to that of the rest of the inbred flock at the Centre.

### CONCLUSIONS

A cholangiocellular carcinoma occurring in a domestic fowl is described, and the structure, incidence and aetiological factors associated with this type of tumour discussed in comparison with similar neoplasia of mammals.

### REFERENCES

BERMAN, C.—(1951) ' Primary Carcinoma of the Liver '. London (H. K. Lewis and Co. Ltd.).

BULLIS, K. L. AND OLSON, C.—(1943) *Amer. J. vet. Res.*, **4**, 382.

CAMPBELL, J. G.—(1945) *J. comp. Path.*, **55**, 308.—(1947) ' Neoplastic Diseases in the Fowl ' *in* ' Diseases of Poultry,' ed. by Blount, W. P. London (Baillière, Tindall and Cox).—(1949) *Brit. J.Cancer*, **3**, 198.—(1955–56) *Proc. roy. Soc. Edinb.*, B, **66**, 111.

COTCHIN, E.—(1956) ' Neoplasms of the Domesticated Mammals, a Review ". Farnham Royal, Bucks (Commonwealth Agricultural Bureau).

EBER, A. AND MALKE, E.—(1932) *Z. Krebsforsch*, **36**, 178.

EDMONDSON, H. A. AND STEINER, P. E.—(1954) *Cancer*, **7**, 462.

FELDMAN, W. H.—(1932) ' Neoplasms of Domesticated Animals '. London (W. B. Saunders).

GOSS, L. J.—(1940) *Cornell Vet.*, **30**, 75.

HEIM, F.—(1931) *Z. Krebsforsch.*, **33**, 76.

JACKSON, C.—(1936) *Onderstepoort J. vet. Sci.*, **6**, 3.

JÖEST, E. AND ERNESTI, S.—(1916) *Z. Krebsforsch.*, **15**, 1.

NORRIS, J. C.—(1936) *Amer. J. Cancer*, **26**, 778.

SCHUCHMANN, K.—(1931) *Arch. Tierheilk*, **63**, 154.

SILLER, W. G. AND OSTLER, D. C.—(1961) *Vet. Rec.*, **73**, 134.